# Correlation between the results of the three methods of RULA, REBA and QEC in assessing the ‎safety and physical health of the employees of a cable manufacturing industry 

**Published:** 2019-08

**Authors:** Reza Hekmatshoar, Ebrahim Taban, Abareshi Fateme, Azmon Tahereh

**Affiliations:** ^*a*^Department of Occupational Health Engineering, Collage of Health, Sabzevar University of Medical Sciences, Sabzevar, Iran.; ^*b*^Department of Occupational Health Engineering, Faculty of Medical Sciences, Tarbiat Modares University, Tehran, Iran.; ^*c*^Master of Science in Persian Literature at Sabzevar Education Directorate, Sabzevar, Iran.

## Abstract

**Background:**

This study is focused safety and physical health on posture analysis of tools RULA, REBA and ‎QEC were used a cable manufacturing company workers. ‎

**Methods:**

The study was conducted on 40 workers engaged in different process of manufacturing. The ‎different activities of the workers were recorded by video and still photography, and these ‎images were used for analysis. Posture analysis tools RULA, REBA and QEC were used. ‎

**Results:**

The results of RULA showed that about 30% of the workers were under high risk level and ‎needed a necessary action immediately. About 37.5% of the workers were under medium risk ‎levels and about 25% of the workers were at lower risk levels. The results of REBA have shown ‎that about 27.5% of the workers were under very high risk levels and required immediate ‎change. About 35% of the workers were at high risk levels and a change is necessary soon, and ‎‎32.5% of the workers were at medium risk levels. According to the QEC method of assessment, ‎it was found that 7.5 of the workers needed no corrective measures. About 35% of the workers ‎needed additional examination and 32.5% of the workers were at high risk and required ‎immediate change. It can be concluded that there are ergonomic deficiencies in the planning ‎and work approaches. A significant proportion of the workers are working in high risk postures. ‎Therefore, the workers are under moderate to high risk of WMSDs. ‎

**Conclusions:**

The current study suggested a suitable implementation of ergonomics interventions program ‎with awareness and training among workers to reduce the risks of WMSD.‎

**Keywords:**

Posture analysis, WMSD, Cable factory ‎

